# The Role of Physical Activity and Rehabilitation Following Hip and Knee Arthroplasty in the Elderly

**DOI:** 10.3390/jcm9051401

**Published:** 2020-05-09

**Authors:** Rocco Papalia, Stefano Campi, Ferruccio Vorini, Biagio Zampogna, Sebastiano Vasta, Giuseppe Papalia, Chiara Fossati, Guglielmo Torre, Vincenzo Denaro

**Affiliations:** 1 Department of Orthopaedic and Trauma Surgery, Campus Bio-Medico University of Rome, 00128 Rome, Italy; r.papalia@unicampus.it (R.P.); s.campi@unicampus.it (S.C.); f.vorini@unicampus.it (F.V.); b.zampogna@unicampus.it (B.Z.); s.vasta@unicampus.it (S.V.); g.papalia@unicampus.it (G.P.); denaro@unicampus.it (V.D.); 2 Department of Movement, Human and Health Sciences, University of Rome “Foro Italico”, 00135 Rome, Italy; chiara.fossati@uniroma4.it

**Keywords:** hip arthroplasty, knee arthroplasty, elderly, physical activity, rehabilitation, physiotherapy

## Abstract

Hip and knee replacement is an effective treatment for symptomatic, end-stage hip and knee osteoarthritis, aiming to relieve pain and restore joint function. Several postoperative rehabilitation protocols and physical activities are proposed in routine clinical practice. However, their effect on clinical outcome and implant revision in patients undergoing joint replacement is still unclear. A systematic review of the literature was performed through a comprehensive search on online databases including Pubmed-Medline, Cochrane central, and Google scholar. We included all the available studies on postoperative physical activity and rehabilitation protocols after total knee and total hip arthroplasty in patients older than 65 years. The primary endpoint was to evaluate the effect of physical activity and rehabilitation on clinical outcome; the secondary outcome was to determine the effect on patients’ quality of life (QoL) and implant survival. Although the heterogeneity of the rehabilitation protocols and outcome measures did not allow to draw definitive conclusions, most studies suggested that aquatic therapy, ergometer cycling, and fast-track protocols have a beneficial effect on muscle strength, gait speed, and main clinical scores after total hip arthroplasty. Similarly, enhanced rehabilitation protocols produced an improvement in primary and secondary outcomes after total knee arthroplasty.

## 1. Introduction

Osteoarthritis (OA) is a major cause of disability in elderly patients. The prevalence of hip and knee OA has been growing over the last decades, being around 25% in the population between 65 and 85 years of age [1]. OA has a considerable impact on patients’ quality of life (QoL), activities of daily living, and general health status. Due to the large number of patients suffering from this condition and the considerable cost of care, OA represents a significant economic burden for healthcare systems [2].

Joint replacement is the only definitive treatment for symptomatic end-stage hip and knee OA, aiming to relieve the pain and restore joint function. Total hip arthroplasty (THA) and total knee arthroplasty (TKA) are usually followed by an intense rehabilitation program focused on muscle strengthening, stretching, range of motion (ROM) recovery, gait rehabilitation, neuromuscular function, and proprioception recovery.

Nowadays, there is an increasing number of elderly people practicing sports and physical activity, whether low-impact (such as cycling, aquafit, golf, swimming) or medium-/high-impact (skiing, running, tennis, dancing, Nordic walking, etc.). Often, patients undergoing hip and knee arthroplasty aim to return to their previous activity level. However, the effect of such activities on the clinical outcome and survival of the implant is still unclear.

Numerous postoperative interventions have been studied, including in-hospital rehabilitation, inpatient rehabilitation, home exercises, tele-rehabilitation, aquatic therapy, and fast-track protocols, but it is still unclear which of these interventions is the most effective following hip and knee arthroplasty in order to achieve a complete functional recovery. Moreover, there is poor evidence about what type of physical activity can be allowed or encouraged without affecting implant survival.

The primary endpoint of this systematic review was to evaluate the impact of physical activity and rehabilitation on clinical objective and subjective outcomes, after TKA and THA. The secondary outcome was to establish the effect of these activities on patients’ QoL and on implant revision rates.

## 2. Materials and Methods

The present systematic review was performed in accordance to the PRISMA guidelines [3] and followed the Cochrane methodology for systematic reviews [4]. The MINORS (methodological index for non-randomized studies) score was used to assess the methodological quality of non-randomized studies [5].

### 2.1. Primary Outcomes

The primary endpoint was to assess the effect of physical activity and rehabilitation on clinical outcome, measured by validated joint-specific objective and subjective clinical measurements. When reported, the considered outcome measures for THA were the WOMAC (Western Ontario and Mc Master University) index, hip abductor strength, Harris Hip Score (HHS), gait speed, UCLA score, Lequesne Hip/Knee score. The considered outcome measures for TKA were the WOMAC index, Lequesne Hip/Knee score, 10 min walking test, walking speed, stair ascending time, knee extensor and flexor power, thigh muscle cross-sectional area, 6 min walking test (6MWT), Knee Society score (KSS), range of motion (ROM), modified gait efficacy scale (mGES), timed up and go (TUG).

### 2.2. Secondary Outcomes

The secondary endpoint was to assess the effect of physical activity and rehabilitation on implant revision rates and self-reported quality of life, using questionnaires.

### 2.3. Search Methods for Identification of the Studies

Online databases, including Pubmed-Medline and Google Scholar, were searched for relevant articles. The search string used was the following: (“sports”(MeSH Terms) OR “sports”(All Fields) OR “sport”(All Fields)) AND (“exercise”(MeSH Terms) OR “exercise”(All Fields) OR (“physical”(All Fields) AND “activity”(All Fields)) OR “physical activity”(All Fields)) AND after(All Fields) AND (“arthroplasty”(MeSH Terms) OR “arthroplasty”(All Fields)) AND (“aged”(MeSH Terms) OR “aged” (All Fields) OR “elderly”(All Fields)).

**Figure 1:**
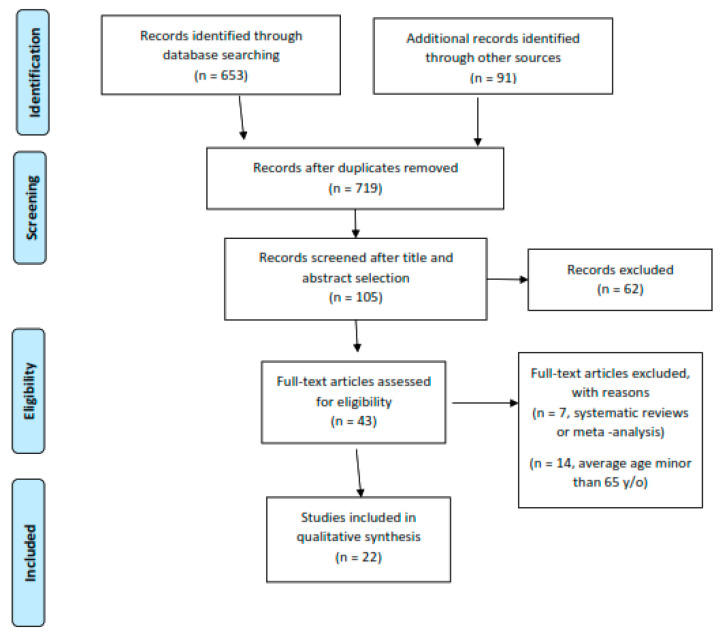
Flow chart of the study inclusion process.

The studies retrieved were firstly screened by title, and if relevant, the whole abstract was red. After a first selection and exclusion of nonrelevant papers, the full text of the potentially eligible articles was retrieved and red by two reviewers, for possible inclusion. Discordant opinions were solved through the consultation of a third reviewer. After the electronic search was completed, the bibliography of the included relevant articles was screened manually to identify further papers, potentially missed in the electronic search. The search process is summarized in the flow diagram in Figure 1. 

### 2.4. Inclusion and Exclusion Criteria 

The studies considered for inclusion were randomized controlled trials (RCT), prospective cohort studies (PCS), retrospective and prospective case–control studies (CCS), longitudinal studies (LS), and cross-sectional studies (CSS). Case reports, reviews, and meta-analyses were excluded. Studies had to report on the postoperative physical activity (intended as early and late physiotherapy, aquatic therapy, and sport activity) in elderly patients who underwent THA and/or TKA. According to the definition of elderly of the WHO, only studies where the average age of the cohorts was superior to 65 years were considered. Studies reporting on both THA and TKA patients needed to present the results of the two groups separately.

### 2.5. Data Collection and Analysis

Data were extracted from the included articles, according to the primary and secondary outcomes considered for the aim of this review. After extraction, generic data concerning the paper were reported in Table 1. For an appropriate presentation of the data, the results were divided on the basis of the type of surgery (TKA or THA).

### 2.6. Risk of Bias Assessment

Given the heterogeneity of the included studies, two different critical appraisal tools were used. For randomized clinical trials, the Cochrane risk of bias assessment tool was applied, providing a grade of risk (low and high risk) of bias for the index study in five elements of the study design (sequence generation, allocation concealment, blinding, incomplete data addressment, and selective reporting). The MINORS score was used for non-randomized studies.

## 3. Results

### 3.1. Results of the Search 

From the electronic and manual search, a total of 744 papers were identified. After the selection process, 43 papers where considered eligible to be included in the study. Fourteen papers were excluded because the average age of the cohort was below 65 years; 7 further papers were excluded for being reviews or meta-analyses. Twenty-two papers (11 RCT, 3 PCS, 6 retrospective CCS, one retrospective CS) were eventually included in the study (Figure 1).

The results of risk of bias assessment are presented in Appendix A and Appendix B.

The included studies reported data on a total of 20,139 patients: 12,818 underwent TKA, while 7321 underwent THA. The average age ranged from 65 to 72.1 years. 

### 3.2. Total Hip Arthroplasty

Six studies reported the results of THA alone and five studies the results of both THA and TKA. Seven studies were RCT, two were retrospective CCS, one was a PCS, and one was a retrospective case series. 

The risk of bias assessment revealed that six of the seven RCT had one or more major methodology flaws, therefore the risk of bias within a single trial was present for these studies (Appendix A). Among non-randomized studies, some showed major limitations, especially concerning patient allocation, blinding, and data collection. The mean MINORS score was 13, indicating a moderate risk of bias (Appendix B). Only one study specified the surgical approach (posterior) [16].

The most frequent primary outcome reported was the WOMAC index, mentioned in four papers [7,8,9,10]. All the included studies showed an improvement in the three subscales (function, pain, and stiffness), with mean values of 13.7, 9.3, and 18.5, respectively. Different types of physical activity and physiotherapy (early and late hydrotherapy [10,13], ergometer cycling [12], intensive physiotherapy addressing specific muscle groups [22]) had a beneficial effect after THA, as measured by the WOMAC score. 

Other studies reported heterogeneous patient-related outcome measures and physical test measures. An improvement in Hip disability and Osteoarthritis Outcome Score – Physical Function Shortform (HOOS-PS) was reported by Winther et al. [15] and by Heiberg et al. [16], after an intensive fast-track treatment and after a walking skill training program, respectively. Two papers reported an increase in HHS values [11,12]. Regarding the physical tests, improvements in hip abductor strength, gait speed, one-legged stance, and 6MWT were reported after intensive aquatic therapy [23] and fast-track gym treatment [22].

Considering the effect of physical activity on quality of life, three papers reported a significant increase of it using the SF-36 questionnaire (mean value 55.6 three months after surgery) [12,13,20]; other authors reported an improvement using the health-related (HR)QoL and EQ-5D questionnaires after fast-track intensive treatment [15,22]. Poor evidence is available about the effect of physical activity on revision rates: Gschwend et al. reported an inferior rate of implant loosening and revisions in active patients [6], while Bauman et al. reported no signs of wear or loosening in patients with high UCLA score 40 months after surgery [27].

The included studies reported on a vast and heterogeneous group of postoperative activities, ranging from high- and low-impact sport activity, to enhanced physiotherapic protocols and early and late hydrotherapy (Table 2). Aquatic therapy, fast-track treatmen,t and leisure physical activity were the most frequently reported activities.

### 3.3. Total Knee Arthroplasty

Nine studies reported the effect of physical activities on TKA alone, and five on both THA and TKA. Out of these, six were RCT, four were retrospective CCS, three were PCS, and one was a retrospective case series. The risk of bias assessment showed that three of the six RCT had one or more major methodological flaws, therefore the risk of bias within a single trial was present for these studies (Appendix A). Among non-randomized studies, some also showed major limitations, especially concerning patient allocation, blinding, and data collection. The mean MINORS score for TKA studies was 13.4, indicating a moderate risk of bias (Appendix B).

Similar to THA, the most frequently reported measure of primary outcome was the WOMAC index, mentioned by six studies [11,12,13,14,24,25]. Differently from the data presented on THA, only four of six studies showed an improvement in the WOMAC index. Only in two of these, the difference was statistically significant. The study by Liebs et al. did not corroborate the use of a cycloergometer after TKA [12]. An early start of hydrotherapy after TKA led to an improved WOMAC at three and six months of follow-up [11,13], but after one year the difference with respect to the control groups disappeared [13,25,26]. In a retrospective CCS by Mayr et al., a significant correlation between sport activity level (high-, medium-, and low-impact) and WOMAC index was reported.

Other patient-related outcome measures analyzed in the reported studies were the Knee injury and Osteoarthritis Outcome Score (KOOS) – Physical Function Shortform (KOOS-PS), KSS, Lequesne Hip/Knee score, and Oxford Knee Score (OKS). An improvement in KSS, KOOS, and OKS was reported after fast-track treatment [15] in patients performing high-activity and high-impact sports [9,14] (though no difference was shown in clinical outcome between these subgroups) [21], after a rehabilitation program targeting quadriceps strength and range of motion [19], and in patients accomplishing moderate-to-high physical activity (UCLA > 6) [27]. Moreover, a wide range of physical tests were reported, including the 6MWT, TUG, sit-to-stand time, knee flexor and extensor power, mGES, gait speed, and thigh muscle cross-sectional area. Aquatic therapy, gait training, muscular strengthening, and enhanced rehabilitation programs had a beneficial effect on these parameters [18,19,24,25], even at long-term follow-up [20].

Considering the effect of physical activity on quality of life, ergometer cycling did not produce significant improvement evaluated with the SF-36 questionnaire [12]. In contrast, early-phase aquatic therapy, enhanced physiotherapic protocols, and fast-track treatment induced beneficial effects according to the SF-36 and EQ-5D questionnaires [9,11,18,21]. 

Considering revision rates and prosthetic wear or loosening, Jones et al. showed that leisure, occupational, and high-intensity activity, measured as MET-hours/week, did increase the risk for revision [8]. Mont et al. reported that high-impact and high-activity sports are not a cause of implant failure (considering clinical and radiographical criteria of the Knee Society rating system), at 4 years of mean follow-up [9]. Similarly, Mayr et al. reported no evidence of wear or loosening, as well as similar revision rates, in patients performing high-impact activity vs. those performing medium- or low- impact activities [14]. Finally, comparing a sportive patient group with an inactive patient group, Valle et al. showed a reduced revision rate in the sport group (15.2% vs. 23.8%) at 12 years of follow-up [25].

## 4. Discussion

THA and TKA are extremely common surgical procedures. However, there is no consensus about the most effective postoperative physiotherapy and physical activities allowing an optimal recovery. The aim of this systematic review was to analyze the current evidence on the role of physical activity and rehabilitation in patients’ clinical outcome after hip and knee arthroplasty.

Aquatic therapy resulted beneficial after THA, with improvements in terms of muscle strength, gait speed, main clinical scores (WOMAC, Lequesne Hip/Knee score, HOOS, HHS), patient satisfaction, and QoL (SF-36, EQ-5D questionnaires). However, an early start of hydrotherapy (6 vs. 14 days after surgery) did not produce further advantages [10,13,23]. Both ergometer cycling and intensified exercise programs resulted beneficial in terms of patient QoL, maximal gait speed, hip abductor muscle strength, and WOMAC index [12]. Interestingly, a supplementary arm and upper body exercise program produced a significant improvement in functional abilities [20]. In general, all protocol focusing on intensified exercises and additional activities and the practice of an adequate sport activity produced a beneficial effect on early and late postoperative recovery and limb function. However, it is unclear whether the same advantages are relevant in term of durability of the prosthetic implant, though it has been reported that a moderate or active lifestyle do not affect implant survival [14,15,26]. Nonetheless, more than 50% of patients undergoing total joint arthroplasty (TJA) do not respect the physical activity guidelines, suggesting that patient education should be improved [17].

It has been demonstrated that fast-track THA protocols are effective in terms of reduced length of stay, patient satisfaction and function [15,28]. However, fast-track is a complex approach, requiring patient preoperative optimization, anesthesia management, systemic pain treatment (nonsteroidal anti-inflammatory drugs (NSAIDs), acetaminophen, short-acting opioids), early mobilization, and physiotherapy [11,27,28,29,30]. It can be really challenging to set up this process in some institutions or for patients with severe comorbidities [31].

Aquatic therapy produced a beneficial effect on the clinical outcome after TKA. The WOMAC index improved in four out of six studies, similarly to SF-36 score, Lequesne Hip/Knee score, and patient satisfaction score [9,13,16,19,20]. Furthermore, also the use of early hydrotherapy (6 days after surgery) showed a beneficial effect similar to the use of NSAIDs [8]. Aquatic therapy improved quadriceps strength, walking speed, stair ascending time. The results of the cycle ergometer are controversial, and this practice is not supported for TKA recovery [12]. It has been suggested that ergometer cycling produces an improvement in strength and proprioception after TKA but that the overload on the knee may induce soft tissue edema and joint effusion, jeopardizing the positive effects [12].

In general, enhanced rehabilitation protocols and fast-track surgery after TKA produced an improvement in primary and secondary outcomes, thus indicating that an early recovery of muscular function and joint proprioception is essential.

The practice of high-impact and high-activity sports is often discouraged by surgeons because of the risk of mobilization and wear of implants’ components. However, the current evidence shows that this practice leads to improved clinical outcomes in elderly patients in term of ROM, KOOS scores, WOMAC index, KSS, pain, and rate of revision [9,14]. Several authors reported that physical activity does not increase the risk of revision. One study suggested that moderate sport activity can improve osteointegration, with a decrease in osteolytic changes and prosthetic loosening [21]. On the basis of these findings, orthopedic surgeons should recommend exercising and participation in moderate and high-level sport activities after joint replacement. However, these data are limited, and there is a need for well-designed studies to draw definitive conclusions on these aspects.

This review has some limitations. First of all, the heterogeneity of the postoperative rehabilitation protocols and activities and the variety of outcome measures reported do not allow to pool the results and perform a statistical analysis. In addition, only one study reported about the surgical technique used and operation details. Nowadays, there are several surgical approaches and different implants that can significantly influence patients’ recovery. However, there is scarce evidence suggesting consistent advantages of one technique over the others on medium- to long-term outcome.

To limit the risk of bias, all papers were evaluated through Cochrane Risk of Bias Assessment and MINORS score, thus enlightening how all RCT were well structured, showing only some incongruity in allocation concealment and blinding. Nevertheless, this seemed not to compromise the quality and the relevance of the results.

## 5. Conclusions

Although the heterogeneity of the rehabilitation protocols and outcome measures do not allow to draw definitive conclusions, most studies suggest that patients over 65 years of age benefit from enhanced physiotherapy protocols, aquatic therapy, and physical activity after knee and hip arthroplasty. The effect of physical activity on implant revision rate and survival remains controversial.

## Figures and Tables

**Table 1:** Details of the included studies.

Study	Type of study, Level of Evidence	Number of Patients	Mean Age (y)	Type of Surgery	Type of Physiotherapy–Exercise–Sport Activity
Gschwend N, et al. Acta Orthop Scand. 2000 [6]	Retrospective clinical study–LOE III	100	65 GROUP A 65 GROUP B	THA	Group A: alpine/cross-country skiing, summer sports (trekking, biking, swimming) Group B: no winter sports
Lavernia CJ et al., J Arthroplasty 2001 [7]	Retrospective	28	68 at the time of surgery	TKA	Physical activity level assessed with UCLA activity score and Charnley class
Jones DL et al. J Rheumatol. 2004 [8]	CCS–LOE III	52	cases 70.5 controls 75	TKA	Leisure and occupational historical/high-intensity activity, measured as MET (metabolic equivalent of task) -hours/wk
Mont MA et al. J Arthroplasty. 2008 [9]	Retrospective CCS–LOE III	148	High-Impact cohort: 66 High-activity cohort: 69 Sedentary cohort: 71	TKA	High-activity: baseball and basketball, gymnastics, hockey; High-impact: aerobics, ice/roller skating, jogging, martial arts, racquetball/squash, rock climbing, skiing (downhill), soccer, tennis (singles)
Giaquinto S et al. Arch Gerontol Geriatr. 2010 [10]	RCT–LOE I	64	Group A: 70.6 Group B: 70.1	THA	Group A: conventional gyms (no-hydrotherapy group) Group B: hydrotherapy group
Giaquinto S et al. Arch Gerontol Geriatr. 2010 [11]	RCT–LOE I	58	Geriatric population, groups matched by age, gender, and body mass index (BMI)	TKA	Group A: conventional gym treatment Group B: hydrotherapy
Liebs TR et al. J Bone Joint Surg Am. 2010 [12]	RCT–LOE I	203 THA 159 TKA	THA Group A: 67.2; Group B 67.2 TKA Group A: 69.9; Group B 69.7	TKA and THA	Group A: No ergometer cycling Group B: ergometer cycling
Liebs TR et al., Arch Phys Med Rehabil. 2012 [13]	RCT–LOE I	280 THA 185 TKA	THA Group A: 66.7; Group B 69.1 TKA Group A: 68.5; Group B 70.9	TKA and THA	Group A: aquatic therapy (pool exercises aimed at training of proprioception, coordination, and strengthening) from the 6th day after surgery Group B: aquatic therapy from the 14th day after surgery
Mayr HO et al., J Arthroplasty. 2015 [14]	Retrospective CCS–LOE III	81	71.8 ± 5.4	TKA	High-impact: alpine skiing, rock climbing, dancing, tennis; Medium-impact: hiking, cross-country skiing, Nordic walking, fitness; Low-impact: aqua fit, golf, cycling, swimming
Winther SB et al. Acta Orthop. 2015 [15]	Retrospective cohort study–LOE III	585 THA 335 TKA	65–66 for THA/TKA primary surgery, 68/67 for revision surgery	TKA and THA	Fast-track (treatment chain)
Heiberg KE et al., Arthritis Care Res. 2016 [16]	RCT–LOE I	60	Training group 70.2 Control group 70.6	THA	Case group: walking skill training program 3–5 months after surgery Control group: usual training
Paxton EW et al., Acta Orthop. 2016 [17]	Prospective comparative study–LOE II	5.678 THA; 11.084 TKA	THA: 68 TKA: 67	THA and TKA	Self-reported minutes of physical activity/week
Taniguchi M et al. J Arthroplasty. 2016 [18]	PCS–LOE II	81	72.1	TKA	Passive knee range of motion (ROM) exercises, strengthening, gait and ADL (activities of daily living) training, cycling with a stationary bicycle
Hiyama Y et al., J Knee Surg. 2017 [19]	Prospective cohort study–LOE II	59	71.7	TKA	standardized rehabilitation program, targeting knee range of motion, pain control, and quadriceps strength
Mitrovic D et al., Clin Rehabil. 2017 [20]	RCT–LOE I	70	Study group: 69.2 Control group: 68.1	THA	Supplementary arm and upper body exercise program to be compared with the standard-rehabilitation program group (upper limb flexibility, range of motion, and muscle strength, along with regular, deep breathing exercises)
Valle C et al., Sportverletz Sportschaden Organ Ges Orthopadisch-Traumatol Sportmed. 2017 [21]	Retrospective CCS–LOE III	130	69.2	TKA	Sport group: trekking, swimming, golf, Nordic walking, skiing No-sport group: without any sport activity
Mikkelsen LR et al., Physiother Res Int J Res Clin Phys Ther. 2012 [22]	RCT–LOE I	44	Intervention group 67.7 Control group 66.8	THA	Fast-track group: rubber band resistance (Thera-Band) and step exercises. Address the muscle groups mostly affected after THA Control group: standard rehabilitation consisting of exercises without external resistance and progression
Rahmann AE et al., Arch Phys Med Rehabil. 2009 [23]	RCT–LOE I	65	Group 1: 70.4 Group 2: 69.4 Group 3: 69	TKA and THA	Group 1: ward physiotherapy treatment each day, following the standard orthopedic clinical pathway; Group 2: aquatic physiotherapy program (30% body weight (BW)); Group 3: water exercise program (10%BW).
Moffet H et al., Arch Phys Med Rehabil. 2004 [24]	RCT–LOE I	77	Standard physiotherapy 68.7 Intensive physiotherapy 66.7	TKA	STANDARD: simple exercises to retrain lower-limb strength (quadriceps, hamstrings, hip abductors, and extensors) and to increase knee mobility, as well as some advice about knee positioning, ice application, and gait retraining; INTENSIVE: 5 components: warm-up, specific strengthening exercises, functional task-oriented exercises, endurance exercises, and cool-down
Valtonen A et al., Arch Phys Med Rehabil. 2010 [25]	RCT–LOE I	50	Training Group 66.2 Control Group 65.7	TKA	Training group: 12-week progressive aquatic resistance training; Control: no intervention
Valtonen A et al., Arch Phys Med Rehabil. 2011 [26]	RCT–LOE I Follow up	42	Training Group 66.2 Control Group 65.7	TKA	Training group: 12-week progressive aquatic resistance training; Control: no intervention
Bauman S et al., Clin J Sport Med. 2007 [27]	Retrospective case series–LOE IV	170 THA 184 TKA	THA 66.4 TKA 68.9	TKA and THA	Physical activity assessed by UCLA activity score

TKA: total knee arthroplasty, THA: total hip arthroplasty, LOE: Level of evidence, CCS: case–control studies, RCT: randomized controlled trials, PCS: prospective cohort studies.

**Table 2:** Types of activity and number of studies in which they were reported.

TYPE OF ACTIVITIES	THA (*n*° of studies)	TKA (*n*° of studies)
Hydrotherapy	3 [1,2,3]	5 [2,3,4,5,6]
Ergometer cycling	1 [7]	2 [7,8]
Intensive physiotherapy		3 [8,9,10]
Fast-track treatment	2 [11,12]	1 [11]
Walking skill training	1 [13]	
Arm/upper body exercise	1 [14]	
Leisure activity (MET-hours/week, minutes/week)	2 [15,16]	4 [15,16,17,18]
Winter sports (alpine skiing, cross-country skiing)	1 [19]	3 [18,20,21]
Summer sports (trekking, hiking, biking, swimming)	1 [19]	3 [18,20,21]
High-impact physical activity (baseball, gymnastics, hockey, basketball, martial arts, football, tennis)		2 [18,20]
Low-impact physical activity (aquafit, golf, cycling, swimming)		2 [18,20]

## Appendix A: Cochrane Risk of Bias Assessment. Low risk (L), High risk (H), Unclear (U).

**Study**	**Sequence generation**	**Allocation Concealment**	**Blinding**	**Incomplete data addressed**	**Free of selective reporting**	**Free of other bias**
Giaquinto et al. [7]	U	L	U	L	L	L
Giaquinto et al. [16]	L	L	L	L	L	L
Liebs et al. [8]	L	L	H	L	L	L
Liebs et al. [9]	L	L	L	L	L	L
Heiberg et al. [6]	H	H	L	L	L	H
Mitrovic et al. [12]	L	L	L	L	L	L
Mikkelsen et al. [10]	U	U	H	U	L	H
Rahmann et al. [13]	L	L	L	L	L	H
Moffet et al. [24]	L	L	L	L	L	L
Valtonen et al. [25]	U	L	H	L	L	L

## Appendix B: MINORS score. The Ideal Scores for Non-Randomized Studies are 16 for Non-Comparative Studies and 24 for Comparative Studies.

**Study**	**SCORE**
Gschwend et al. [6]	19
Jones et al. [8]	20
Mont et al. [9]	12
Mayr et al. [14]	17
Winther et al. [11]	11
Paxton et al. [17]	12
Taniguchi et al. [18]	15
Hiyama et al. [19]	15
Valle et al. [21]	11
Valtonen et al. [26]	11
Bauman et al. [15]	10
